# Research into the Health Benefits of Sprint Interval Training Should Focus on Protocols with Fewer and Shorter Sprints

**DOI:** 10.1007/s40279-017-0727-x

**Published:** 2017-04-08

**Authors:** Niels B. J. Vollaard, Richard S. Metcalfe

**Affiliations:** 1 Faculty of Health Sciences and Sport, University of Stirling, Stirling, FK9 4LA UK; 2 School of Sport, Ulster University, Northern Ireland, UK

## Abstract

Over the past decade, it has been convincingly shown that regularly performing repeated brief supramaximal cycle sprints (sprint interval training [SIT]) is associated with aerobic adaptations and health benefits similar to or greater than with moderate-intensity continuous training (MICT). SIT is often promoted as a time-efficient exercise strategy, but the most commonly studied SIT protocol (4–6 repeated 30-s Wingate sprints with 4 min recovery, here referred to as ‘classic’ SIT) takes up to approximately 30 min per session. Combined with high associated perceived exertion, this makes classic SIT unsuitable as an alternative/adjunct to current exercise recommendations involving MICT. However, there are no indications that the design of the classic SIT protocol has been based on considerations regarding the lowest number or shortest duration of sprints to optimise time efficiency while retaining the associated health benefits. In recent years, studies have shown that novel SIT protocols with both fewer and shorter sprints are efficacious at improving important risk factors of noncommunicable diseases in sedentary individuals, and provide health benefits that are no worse than those associated with classic SIT. These shorter/easier protocols have the potential to remove many of the common barriers to exercise in the general population. Thus, based on the evidence summarised in this current opinion paper, we propose that there is a need for a fundamental change in focus in SIT research in order to move away from further characterising the classic SIT protocol and towards establishing acceptable and effective protocols that involve minimal sprint durations and repetitions.

## Key Points

**Table:** 

Over the past decade, aerobic fitness adaptations and health benefits following sprint interval training (SIT) have received much attention. However, the most commonly used SIT protocol, involving 4–6 repeated ‘all-out’ 30-s cycle sprints, is very demanding and not as time efficient as often suggested.
Recent studies demonstrate that both the number of sprint repetitions and the sprint duration of SIT protocols can be reduced (to a point) without attenuating the associated health benefits.
Based on the evidence that we present in this article, we contend that the focus of SIT research should be moved towards establishing acceptable and effective protocols that involve minimal sprint durations and repetitions.

## Background

Addressing the negative consequences associated with the high prevalence of physical inactivity in the general population [1] is one of the main public health challenges of current-day society. As lack of time has consistently been identified as one of the main perceived barriers preventing sedentary individuals from becoming, and remaining, physically active [2–4], this has led to the question of whether a subgroup of sedentary individuals would be more willing or able to reap the benefits of regular exercise if alternative, more time-efficient options were available. A one-size-fits-all approach to exercise recommendations may not suit all individuals, and alternative/adjunct interventions need to be identified in order to overcome this problem. Therefore, over the past decade there has been increasing interest in the use of exercise interventions that enable health benefits with shorter exercise times by increasing exercise intensity [5, 6].

**Fig. 1:**
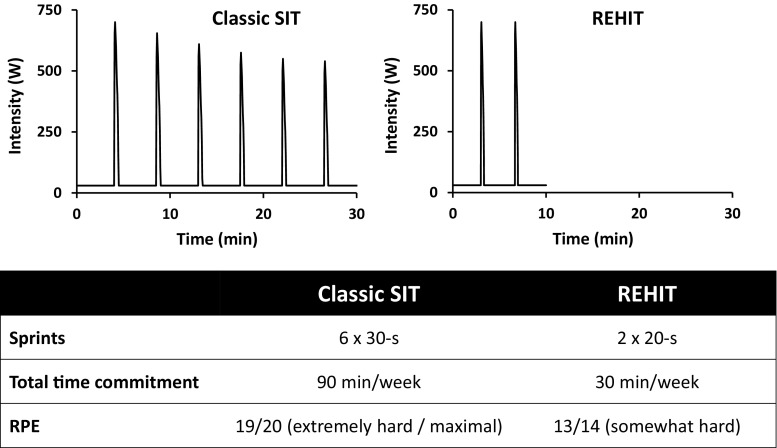
Comparison of the classic SIT protocol with the novel REHIT protocol. *SIT* sprint interval training, *REHIT* reduced-exertion high-intensity interval training, *RPE* rating of perceived exertion

As high exercise intensities cannot be maintained for extended periods of time, the logical approach has been to develop interval protocols alternating repeated bouts of (sub)maximal exercise (high-intensity interval training [HIIT]) or supramaximal exercise (sprint interval training [SIT]) with recovery intervals. The time spent performing high-intensity intervals in common HIIT protocols (approximately 10–16 min) [7, 8] is larger than that in common SIT protocols (approximately 2–3 min) [9]; therefore, in theory, the latter have a greater potential to provide a time-efficient alternative or addition to current recommendations based on moderate-intensity continuous training (MICT). The most commonly studied SIT protocol progresses from four repeated 30-s Wingate sprints at the start of a 2- to 8-week programme to six sprints towards the latter stages (hereafter termed ‘classic’ SIT) (Fig. 1). In a recent meta-analysis of the effects of SIT on maximal aerobic capacity ($\dot{V}$O_2_max), this specific protocol was used by more than half of all included studies [10]. However, the classic 4–6 × 30-s SIT protocol is extremely fatiguing and is not actually that time efficient; including a warm-up and 4 min recovery following sprints, the total time commitment is approximately 30 min per training session towards the end of the training programme. The resulting total time commitment of approximately 90 min per week is greater than the current recommendations for vigorous-intensity continuous exercise of 75 min per week [11]. Thus, considering the proclaimed aim of SIT to provide a time-efficient alternative/adjunct to current exercise recommendations, it is surprising that relatively little attention has been given to investigating whether various SIT protocol parameters (e.g. number of sprint repetitions, training frequency, sprint duration, intensity) can be modulated to achieve beneficial cardiometabolic adaptations with a lower time commitment and reduced perceived exertion. As each of these protocol parameters will impact on the likelihood of sedentary individuals adopting and adhering to an SIT intervention, this is an important area of research. In this paper, we present an overview of the growing body of recent research that suggests the classic SIT protocol is unnecessarily long and strenuous, and make a case for changing the focus of SIT research towards protocols that are shorter, easier and potentially even more effective.

## What is the Evidence-Base for the Design of the Classic Sprint Interval Training (SIT) Protocol?

In the 1980s and 1990s, several training studies investigated the effects of SIT protocols with 8–10 repeated 30-s Wingate sprints on a range of physiological outcomes, including maximal glycolytic and mitochondrial enzyme activity [12, 13], purine metabolism [14], pulmonary and muscle gas exchange [15], muscle metabolism and ion regulation [16], muscle buffering capacity [17], and erythrocyte characteristics [18]. Although never stated as a main aim, several of these studies provided evidence that regularly performing 8–10 repeated Wingate sprints improves $\dot{V}$O_2_max [13, 15, 16, 18], which has consistently been shown to be the strongest predictor of future morbidity and mortality [19–22]. However, as performing 8–10 repeated Wingate sprints is associated with severe fatigue, and, including recovery time, takes ≥35 min per training session, these protocols were never proposed to be of practical use for sedentary individuals. Interestingly however, Allemeier et al. [23] had by then already demonstrated that $\dot{V}$O_2_max can be improved by approximately 14% with as little as three repeated Wingate sprints per training session, but this finding received little attention.

The classic SIT protocol incorporating up to six repeated 30-s Wingate sprints was first used in a study by Barnett et al. [24], who reported an 8% increase in $\dot{V}$O_2_max and a 42% increase in maximal citrate synthase activity following 8 weeks of SIT. This protocol, with minor modifications, was subsequently used by Gibala’s group at McMaster University in a series of seminal studies investigating the aerobic adaptations associated with classic SIT [5, 25–27]. None of these studies provided a specific justification for the use of 4–6 repeated Wingate sprints, and the authors did not comment on whether the protocol was developed to optimise a specific training stimulus or to maximise a hypothesised mechanism of adaptation. Nonetheless, the classic SIT protocol was shown to be effective at inducing peripheral and whole-body aerobic adaptations, and the majority of subsequent studies investigating aerobic adaptations and/or health benefits of SIT have since used this protocol or minor modifications thereof. To our knowledge, no publications have attempted to justify why performing 4–6 × 30-s Wingate sprints would be an optimal SIT protocol, i.e. many researchers appear to have opted to persist with a protocol that works. We cannot but conclude that the number and duration of sprints used in the classic SIT protocol has been mostly arbitrary.

## How Effective is the Classic SIT Protocol?

There is a large body of evidence to support the efficacy of the classic SIT protocol for improving a variety of important health parameters, including $\dot{V}$O_2_max [28–33], insulin sensitivity [33–35], blood pressure [33, 36], cardiovascular function [37] and body composition [33, 38, 39]. However, in order for any exercise intervention to be recommended to the general public for improving or maintaining good health, the benefits of the intervention will need to be at least as good as those associated with current exercise recommendations. Although several studies have directly compared SIT protocols with MICT, interestingly the MICT condition often involves exercise intensities and durations that exceed current exercise recommendations [26, 32, 40, 41]. Despite this, SIT protocols tend to compare favourably with MICT; meta-analyses have concluded that SIT is as good as or better than MICT at improving, for example, $\dot{V}$O_2_max [9, 42, 43] and insulin sensitivity [44] (although it should be noted that some of these have included both HIIT and SIT studies in the analysis [9, 44]). However, while experimental data thus clearly support the efficacy of SIT (i.e. producing beneficial results in laboratory studies), the effectiveness of SIT (i.e. producing beneficial results under ‘real-world’ conditions) is often questioned [45–47]. In recent years, it has been argued that the high exercise intensities in SIT protocols may make SIT “unsafe, unpractical or intolerable for general populations” [48]. Detractors of SIT propose that the strenuous nature of supramaximal sprint exercise will result in negative affect and, consequently, low uptake of and adherence to SIT [46]. However, it is important to bear in mind that no studies have produced data to support the suggestion that medium- to longer-term adherence to SIT will be low. Nonetheless, it seems reasonable to hypothesise that members of the general public who currently fail to achieve the MICT-based recommendations will not consider performing approximately 30 min of classic SIT to be an attractive alternative [49].

## Do Proposed Mechanisms Support the Use of the Classic SIT Protocol?

It is generally accepted that greater volumes/higher intensities of MICT will lead to more pronounced cardiometabolic adaptations [50–52], therefore it is tempting to assume that performing more and/or longer supramaximal sprints will also enhance the cardiometabolic adaptations associated with SIT. However, this cannot be a foregone conclusion as, unlike with small volumes of MICT, the disturbance of homeostasis following just a single supramaximal cycle sprint is already rapid and severe. An understanding of the specific stimuli and subsequent signalling pathways responsible for the various adaptations associated with SIT would be helpful in identifying protocols that are more time efficient and less strenuous, but progress to date has been limited. The initial stimuli could either involve factors that would be expected to lead to greater adaptations with more/longer sprints, such as energy turnover or time spent at high intensity, or factors that may be similar with fewer/shorter sprints, such as peak power generation [53], maximal activation of metabolic pathways, or maximal increases in specific metabolites or signalling molecules. Furthermore, cardiometabolic adaptations could be initiated by either central factors (e.g. changes in heart rate, stroke volume, blood flow, plasma volume) or peripheral factors (e.g. glycogen depletion, increased intramyocellular [Ca^2+^] and [AMP], ryanodine receptor fragmentation and sarcoplasmic reticulum Ca^2+^ leaking [54], etc.). Although $\dot{V}$O_2_max is generally considered to be limited by central factors [55], recent data have challenged this view [56], and several authors have proposed that SIT may increase $\dot{V}$O_2_max due to increased mitochondrial density [31, 41, 43, 57, 58]. Similarly, changes in insulin sensitivity may be due to adaptations within the muscle [59], but could also be due to, for example, improved delivery of insulin and glucose to skeletal muscle due to cardiovascular adaptations [60].

Potential mechanisms have been investigated in HIIT studies [7, 58, 61, 62], but as there is a several-fold difference in exercise intensity between SIT and HIIT, it remains unclear whether such information is relevant for SIT protocols. Few authors have provided detailed hypotheses about which stimuli may be responsible for specific adaptations with SIT, but hypotheses on peripheral mechanisms appear to be more prevalent [6, 54, 63, 64]. Gibala’s group proposed that, similar to MICT, cardiometabolic adaptations to SIT are secondary to activation of upstream kinases, including 5′-adenosine monophosphate-activated protein kinase (AMPK) and p38 mitogen-activated protein kinase (MAPK), which subsequently activate the proposed ‘master regulator’ of mitochondrial biogenesis and function, peroxisome proliferator-activated receptor gamma coactivator (PGC)-1α [63, 65]. There is sufficient evidence to suggest that these pathways are indeed activated with repeated supramaximal sprints [30, 65, 66], and that mitochondrial density rapidly increases in response to SIT [5, 25, 30]. We have subsequently proposed that this may be due to rapid glycogen depletion and associated release of glycogen-bound AMPK [66, 67]. Glycogen depletion during repeated Wingate sprints is attenuated by the third sprint [68], and the increase in activation of various signalling kinases and transcriptional regulatory proteins in response to a single 30-s Wingate sprint [69–72] is indeed similar compared with multiple sprints [65, 73]. We therefore hypothesised and demonstrated that protocols with fewer and shorter sprints result in similar acute signalling responses [66] and chronic adaptations [67, 74] compared with the classic SIT protocol. However, our recent findings that performing single 20-s Wingate sprints three times per week is not a sufficient stimulus for improving $\dot{V}$O_2_max [75, 76] does not provide support for the hypothesis that AMPK-activation following glycogen depletion is responsible for causing increases in $\dot{V}$O_2_max.

To date, the limited amount of available data neither supports nor refutes that the classic 4–6 × 30-s SIT protocol will lead to more pronounced activation of potential signalling pathways involved in any of the cardiometabolic adaptations to SIT compared with shorter/easier protocols. It is entirely plausible that the severe disruption of homeostasis associated with supramaximal exercise rapidly ‘saturates’ the signalling response and that regularly performing just a few brief supramaximal sprints is sufficient to gain the desired health benefits.

## Evidence to Support the Efficacy of Fewer and/or Shorter Sprints

A growing body of evidence supports that performing fewer and/or shorter sprints does not impair the cardiometabolic adaptations associated with SIT (Table 1). Hazell et al. [53] directly compared the impact of reducing sprint duration in the classic SIT protocol from 30 to 10 s, and reported similar increases in $\dot{V}$O_2_max with the 10-s protocol. This finding was supported a few years later by Zelt et al. [41], who reported no significant difference in $\dot{V}$O_2_max response to the classic SIT protocol with 30-s sprints (4%) and a modified protocol with 15-s sprints (8%). These findings are important as the duration of supramaximal sprints has a substantial impact on perceived exertion; the strong contribution of phosphocreatine hydrolysis to energy demands during the first approximately 10 s of a 30-s Wingate sprint means that fatigue during this phase is relatively low, whereas the gradual switch to glycolysis as the predominant energy source [68] is associated with severe and progressive fatigue during the latter stages of the sprint.

**Table 1:** Overview of studies investigating the effects of cycling-based SIT protocols involving shorter and/or fewer sprints than used in the classic 4–6 × 30-s Wingate protocol

Study	Subjects	Training parameters					Outcome
		Duration (weeks)	Frequency (sessions/week)	Sprint duration (s)	Sprint repetitions	Total training time per session (min)	
Shorter sprints							
Hazell et al. [53]	48 M (ra)	2	3	10 vs. 30	4–6	11 vs. 21 vs. 23	No significant difference between increases in $\dot{V}$O_2_max with 30-s sprints (9.3%), 10-s sprints with 4-min recovery (9.2%), or 10-s sprints with 2-min recovery (3.8%)
Zelt et al. [41]	36 M (ra)	4	3	15 vs. 30	4–6	35	No significant difference between the increases in $\dot{V}$O_2_max with 30-s sprints (5.3%) or 15-s sprints (7.4%)
Fewer sprints							
Allemeier et al. [23]	17 M (ra)	6	2.5	30	3	41.5	13.5% increase in $\dot{V}$O_2_max
Ijichi et al. [77]	20 M (ra)	4	2.5 vs. 5	30	3 vs. 6	21.5 vs. 103	No significant difference between the increase in $\dot{V}$O_2_max with 3 sprints 5 times/week (13.9%) or 6 sprints 2.5 times/week (8.4%)
Shorter and fewer sprints							
Songsorn et al. [75]	30 M/F (sed/ra)	4	3	20	1	0.3	No significant increase in $\dot{V}$O_2_max
Songsorn et al. [76]	10 M/F (sed/ra)	4	3	20	1	4.3	No significant increase in $\dot{V}$O_2_max
Metcalfe et al. [67]	29 M/F (sed)	6	3	20	2	10	12.7% increase in $\dot{V}$O_2_max; 28% increase in Si in men
Metcalfe et al. [74]	35 M/F (sed)	6	3	20	2	10	9.6% increase in $\dot{V}$O_2_max; trend toward 10% decrease in OGTT insulin AUC
Ruffino et al. [87]	16 M (T2D)	8	3	20	2	10	7.3% increase in $\dot{V}$O_2_max; 4% decrease in MAP, no significant change in Si
Gillen et al. [78]	14 M/F (o/o sed)	6	3	20	3	10	12% increase in $\dot{V}$O_2_max; 7% decrease in MAP, 8% decrease in CGM AUC
Gillen et al. [57]	25 M (sed)	12	3	20	3	10	19% increase in $\dot{V}$O_2_max; 53% increase in Si, changes similar to MICT

*AUC* area under the curve, *CGM AUC* area under the curve for 24-h continuous glucose monitoring, *F* female, *M* male, *MAP* mean arterial pressure, *MICT* moderate-intensity continuous training, *OGTT* oral glucose tolerance test, *o/o* overweight/obese, *ra* recreationally active, *sed* sedentary, *Si* insulin sensitivity, *SIT* sprint interval training, *T2D* type 2 diabetes, $\dot{V}$ *O*_*2*_ *max* maximal aerobic capacity

Similar to reducing sprint duration, reducing the number of sprint repetitions will also decrease the perceived exertion associated with SIT. As mentioned in Sect. 2, in the early 1990s Allemeier et al. [23] demonstrated robust improvements in $\dot{V}$O_2_max following a protocol involving three repeated 30-s Wingate sprints; however, this protocol involved 20 min of passive recovery following each sprint, negating its potential time efficiency. More recently, Ijichi et al. [77] also used long recovery intervals (10 min) in between three repeated 30-s Wingate sprints, and confirmed the potential of a low number of sprint repetitions to improve $\dot{V}$O_2_max (+14% following 4 weeks of training).

In our own laboratory, we have demonstrated that 6 weeks of 3-weekly 10-min SIT sessions involving just two 20-s Wingate sprints (termed ‘reduced-exertion HIIT’, or REHIT) (Fig. 1) is sufficient to improve $\dot{V}$O_2_max by 10–13% [67, 74]. The ability of the REHIT protocol to improve oral glucose tolerance test-derived measures of insulin sensitivity was unclear, with a significant improvement in the Cederholm Index in men in one study [67], but only a trend towards a 10% reduction in insulin area under the curve (irrespective of sex) in the other study [74]. Gibala’s group [57, 78] subsequently modified the REHIT protocol to include a third 20-s sprint. In an initial study, they confirmed the increase in $\dot{V}$O_2_max (+12%) and reported a 7% decrease in mean arterial pressure [78]. Glycaemic control, determined through 24-h continuous glucose monitoring, significantly improved in men but not women [78]. In a follow-up study, Gibala’s group demonstrated that the increase in $\dot{V}$O_2_max does not plateau after 6 weeks (+12% after 6 weeks and +19% after 12 weeks), and that the increase in insulin sensitivity in response to REHIT (+53%) is not significantly different compared with the increase with MICT (34%) [57].

The strongest evidence in favour of the efficacy of shorter/easier SIT protocols comes from our recent meta-analysis in which we examined the modifying effect of the number of sprint repetitions in an SIT session on the increase in $\dot{V}$O_2_max [10]. A surprising finding of this study was that this effect may be negative, i.e. increasing the number of sprint repetitions may actually decrease the improvement in $\dot{V}$O_2_max. Although the magnitude-based inference of this effect was ‘possibly small’, and therefore further research is required to provide a definitive answer to the question as to whether performing more sprints is worse, this question is irrelevant for the practical implications of our finding; performing more sprints was clearly not *better* for improving $\dot{V}$O_2_max. The logical question then is whether regularly performing just a single supramaximal sprint will be sufficient to improve $\dot{V}$O_2_max; however, in two recent studies we have provided initial data to suggest that this is not the case [75, 76]. It appears that repeating sprints is required for training to be effective. However, to date, all the available evidence suggests that SIT protocols with fewer (two to three) and shorter (10–20 s) sprints are as good as or better than the classic SIT protocol at improving important health markers.

## Implications of Evidence in Favour of Shorter SIT Protocols

In recent years, the focus of research investigating time-efficient alternatives to current exercise recommendations has shifted away from the classic SIT protocol towards HIIT protocols. Gibala’s HIIT protocol involving 10 repeated 1-min sprints at approximately 90% of maximal heart rate interspersed with 1 min recovery [7, 79, 80], and Wisløff’s protocol involving four repeated 4-min sprints at 90% of maximal heart rate interspersed with 3 min recovery [8, 81, 82], are associated with promising results in sedentary individuals and patient populations, and, due to the lower exercise intensities, are proposed to be safer and more likely to be adhered to. However, similar to the classic SIT protocol, these HIIT protocols are not actually very time efficient (25–40 min per training session), and, although (sub)maximal sprints are clearly less strenuous compared with Wingate sprints, the increased sprint duration and number of repetitions result in substantial progressive fatigue and negative affective responses [80, 83, 84]. In contrast, the newly developed REHIT protocol takes no more than 10 min per training session to complete and is associated with acceptable session ratings of perceived exertion [67, 74]. In our experience, the sweat-response to this protocol is low, which removes the need for changing into exercise clothes or having a shower after exercise; our research participants tend to do exercise in their regular clothes. Furthermore, although the need for ‘specialist’ exercise equipment has been raised as a barrier to implementing SIT [85], a recent study has shown that a stair-running-based REHIT protocol can be effective [86]. Furthermore, developing cheap exercise bikes for the use of SIT protocols is not limited by technical difficulties but rather by the issue of supply and demand. Moreover, unlike HIIT, SIT does not require pre-intervention tests to establish appropriate exercise intensities, nor does it require equipment to monitor the prescribed intensity. The shorter exercise session duration (10 min) could facilitate cost-effective use of SIT-enabled stationary bikes in gyms, schools or at the workplace. Thus, shorter, easier SIT protocols have the capacity to remove many of the common barriers that prevent people from adopting and adhering to regular structured exercise [2–4]. We propose that such protocols are particularly well-suited as a primary prevention exercise routine for sedentary individuals. Although the safety of SIT protocols has often been questioned (without data to support this argument), we recently observed no adverse events during an 8-week REHIT intervention in 16 middle-aged overweight/obese, prehypertensive, type 2 diabetes patients [87]. Although this does not ‘prove’ the safety of SIT (safety is a difficult concept to demonstrate experimentally), it does provide some tentative indirect support for SIT to be a safe intervention in sedentary but disease-free individuals. However, more research should address the safety of supramaximal exercise in various populations.

## Conclusions

In a recently published debate on the pros and cons of HIIT as a public health strategy [45], the proponent of HIIT conceded that “no one is proposing Wingate-based SIT as a strategy to impact public health”; however, based on the information presented in this paper, we do precisely this. A growing body of evidence demonstrates the efficacy of SIT protocols with fewer (as little as two repetitions) and shorter (10–20 s) sprints, thus removing many of the proposed barriers to SIT as a feasible intervention for reducing the risk of noncommunicable diseases in the general population. Limiting sprint repetitions and duration makes SIT shorter and easier without attenuating the associated health benefits. This firmly establishes these novel protocols as viable alternatives to current MICT-based recommendations. We contend that research into the health benefits of SIT requires a change of focus—away from further characterising the classic SIT protocol incorporating 4–6 × 30-s Wingate sprints and towards establishing acceptable and effective protocols that involve minimal sprint durations and repetitions.
